# High-altitude pregnancy adaptation: evidence from a Himalayan population in Leh

**DOI:** 10.1098/rstb.2024.0396

**Published:** 2025-08-21

**Authors:** Sara L. Hillman, Padma Dolma

**Affiliations:** ^1^ Institute for Women's Health, University College London Faculty of Population Health Sciences, London, UK; ^2^ Department of Obstetrics, Sonam Norboo Memorial Hospital, Leh, Jammu and Kashmir, India

**Keywords:** birth weight, Ladakh, hypoxia

## Abstract

Leh, Ladakh, is situated at an altitude of more than 3500 metres above sea level (masl) and serves as a natural hypoxic environment for studying the effects of a low-oxygen environment on pregnancy. Over a 10 year collaboration, pregnant women in Leh were recruited and studied, alongside their partners and offspring at birth. The aim was to better elucidate underlying mechanisms that might both protect from, as well as predispose to, poor fetal growth. Phenotypic data were analysed alongside genomic data generated by the Infinium™ Global Screening Array-24 BeadChip. Patient records from low-altitude sites (Bylakuppe 850 m a.s.l) and Delhi (200 m) were compared with data from Leh. A cohort of 318 pregnant women in Ladakh were studied, with an average birthweight for those born at term of 3.15 kg. Compared with women who delivered an appropriate for gestational age (AGA) baby in Leh, those who delivered a small for gestational age (SGA) (<10th birth weight centile) were statistically older and lighter and had smaller maternal uterine artery diameters at 18−22 weeks of gestation. This difference was not maintained between AGA and SGA babies at low altitude in the Delhi cohort. Genetic analysis of Ladakh babies suggested a genetic history most closely related to neighbouring Tibeto-Burman-speaking populations and birth weight analysis replicated previous identified SNPs related to metabolic, skeletal and height genes, offering novel avenues of investigation.

This article is part of the discussion meeting issue ‘Pregnancy at high altitude: the challenge of hypoxia’.

## 1. Introduction

 Carrying a healthy, well grown fetus to term under these conditions is an ultimate demonstration of successful adaptation to a naturally hostile, hypoxic environment. It is well established that in certain HA populations, birth weight decreases with altitude [1–3]. However, birthweight seems less affected in populations who have resided at HA for many generations [4,5]. For example, in La Paz, Bolivia [3], 600 m a.s.l.), native Andean babies are born heavier than their European counterparts [6]. Similarly, babies born to Tibetan women at altitudes above 3000 m a.s.l. are more than 500 g heavier than those born to native lowland Han Chinese [7]. These findings support a HA adaptive mechanism influencing birth weight.

A genetic adaptation is an obvious candidate, especially given the relationship to long ancestry and protection. Genetic studies of birth weight at HA have primarily focused on maternal genotypes. The maternal *PRKAA1* gene locus (coding for AMPK, a central regulator of cellular energy metabolism) has been associated with birth weight and maternal uterine artery (UtA) diameter in HA Andean residents [8].

Leh, Ladakh is situated more than 3500 m a.s.l., making it a relevant HA site. It has a long and culturally significant history within India, forming part of the trade corridor between South Asia and the Tibetan plateau. 'Ladakh’ is the Persian spelling of the term ‘La-dvags’, which in Tibetan means ‘land of high passes’ [9]. Situated in a strategically important position, at one of the highest points populated by humans in the Himalayas, the Ladakhi population makes for interesting study of the effects of altitude on human physiology.

However, unlike other HA populations, the Ladakhi population remains relatively understudied. They share some elements of ancestral history with neighbouring Tibetans [10] and birth weights from this population have been reported. These data support a protective influence of Tibetan ancestry on birth weight, but they failed to confirm the same protective effect for Ladakhi offspring [11]. However, these data are now more than 30 years old.

The development of new therapeutics and treatments is challenging and costly related to reproductive health and childbirth issues, particularly as there are both a pregnant woman and her unborn fetus that need to be considered. Furthermore, given the evidence that many adverse pregnancy complications disproportionally affect low- and middle-income populations [12], research in areas of higher need allows for better development of contextually specific strategies. Investigation of fetal growth and development is a challenging and complex problem and there are currently limited strategies to treat poor growth in the womb. One of the mainstays remains delivery of the fetus to avoid the consequences of extreme poor growth (stillbirth) but this does not alleviate the problem of being born growth-restricted. The impact of growth restriction on the individual can be felt in both the short and long term, with protracted neonatal and adolescent issues [13,14] as well as long-term increased cardio metabolic risk [15,16].

There are recognized maternal and fetal risks for growth restriction [17] but environment—both the intrinsic womb and the external physical world—also plays a role. Altitude, like climate, may affect the ability of a woman’s body to make the necessary changes to support a healthy pregnancy. It is probable that in adverse environments there is a trade-off between maternal and fetal health but that this trade-off may be tailored to deal with the specific adverse stimulus. Normal pregnancy physiology requires the development of a maternal–fetal interface and a placenta that supplies the fetus with the necessary oxygen and nutrients to grow and develop. Conventional placentation theory describes a relatively hypoxic early intrauterine environment that rapidly responds to increased oxygenation through spiral artery dilatation and enhanced blood flow through the placental circulation [18]. Poor growth and pre-eclampsia features include an ischaemic or ‘hypoxic’ presentation within the placenta, presenting as oxidative stress [19]; however, placentas from labouring women, sampled at HA, showed little or no oxidative stress [20]. This suggests adaptation or amplification of placental hypoxia may be a biologically significant mechanism that protects birthweight at HA.

Conditions that reduce fetal oxygen delivery (such as significant maternal cardiac disease) are associated with lower birthweight [21]. Experimental models of oxygen-sensing dysregulation can recapitulate placental failure [22], supporting the idea that external hypoxic environments may provide a natural experimental model for the study of fetal growth restriction. However, it remains important to recognize that the drivers of normal fetal growth may not be directly inversely interchangeable with the causes of pathologically poor growth at altitude and thus careful interrogation and description of the relevant populations is essential.

The aim of this work was to establish the detailed maternal physical characteristics and fetal genotypes associated with fetuses born at an appropriate size compared with those born small for gestation at HA. Furthermore, we hoped to establish whether any differences seen were unique to this environment or could be reproduced in low-altitude settings. The ambition of the study was to generate new insights about the Leh pregnant population that could be extrapolated to other HA research populations, while highlighting important unanswered questions and more unique population aspects that might have clinical relevance related to fetal growth.

## 2. Methods

Over the course of 10 years, work was carried out to facilitate the study of pregnant populations in Leh, Ladakh. This was performed in collaboration and with support from staff at the local hospital in Leh, Sonam Norboo Memorial Hospital (under the leadership of the Chief Obstetrician, Dr Padma Dolma) and through support from University College London (Principal Investigator Dr Sara Hillman, with Professor Hugh Montgomery, Professor David Williams and Dr Dalvir Kular), the Royal College Surgeons Ireland (Professor Cavalleri, Sushil Bhandari and Edmund Gilbert), the Institute of Genomics and Integrative Biology (Professors Mitali Mukerji and Bhavana Prasher) and the All India Institute for Medical Sciences (Professors Vandana Jain and Vatsla Dadhwal). Funding was received from UCL and the Wellcome Trust SEED fund (WT109862/Z/15/Z/WT).

Ethical permissions were granted for the study by the Office of the Chief Medical Officer Leh on the 3rd August 2016 and from the Indian Health Ministry’s Screening Committee on the 7th September 2016, as well as from the All India Institute for Medical Sciences and the University College London Research Ethics Committee (3634/002).

Before commencing the study, an audit was undertaken to update information pertaining to birthweight and other pregnancy outcomes in Leh, to better inform the sample sizes required. Data were collected from paper hospital records and death and maternity delivery physical records were reviewed by a single operator over a 1 year period. Gestational age was confirmed from records and to assist with assessing the best approach to recording birthweight (actual weight or birthweight centiles that can take into account gestational age born and can be more useful in identifying growth-restricted individuals).

A similar audit over the same time period was undertaken at a low-altitude (LA) location (Tso Jhe Khangsar Hospital in Bylakuppe South India) situated at about 850 m a.s.l., identified owing to the presence of a Tibetan population residing at this location.

Next, over a 2 year period (February 2017–January 2019), pregnant women and their partners were recruited to the High Altitude and Pregnancy Study (HAPS) from Sonam Norboo Memorial Hospital, Leh from antenatal clinics and the ultrasound department. In parallel, pregnant women were also recruited and studied in the same way, from AIIMS, New Delhi. Geographical ancestry was recorded for more than three generations for both parents and an extensive family, obstetric and medical history was recorded and has been previously reported [23]. Pregnant women and their partners consented, and blood samples were taken and stored for future analysis. Maternal UtA diameter at was measured at 18−22 weeks of pregnancy, in a standard manner by the same operator.

After birth, mode of delivery and neonatal characteristics (sex, weight, head circumference, crown-heel length and APGAR score) were recorded. Phenotype data were analysed using logistical regression methods. Explanatory variables were assessed as significant at the 5% level and sensitivity analysis was performed on the final multivariable model. Cord blood was sampled after delivery and stored; genomic DNA was extracted at the Institute of Genomics and Integrative Biology, New Delhi and samples processed using the Infinium™ Global Screening Array-24 BeadChip platform.

Genotype assignment was performed using Illumina’s Genome Studio® Version 2.0 (https://emea.support.illumina.com/downloads/genomestudio-2-0.html). Genotypes of Ladakhi individuals were combined with reference individuals from surrounding populations who were identified from the Human Genome Diversity project [24].

Principal components were calculated using PLINK [25] and genetic clustering was performed using ADMIXTURE v.1.2 [26]. Results were further interrogated by applying *f*-statistics [27] to test the strength of evidence that a Ladakh individual is admixed between pairs of neighbouring populations.

Genome-wide association tests were conducted using 601 887 genotyped SNPs. Using the linear command in PLINK v.1.9, linear regression was performed. An additive genetic model that adjusted for fetal sex and the first four principal components obtained from the genome-wide SNP data, were used.

## 3. Results

Audit data including Leh birth records for 380 infants were reviewed and it was possible to confirm gestational age in more than 90% of these cases. In babies born at a term gestation (>37 weeks), the average birthweight was 3.04 kg, with 41 of the 380 infants (11%) found to be born pre-term with an average birthweight of 2.11 kg. If using gestational age to customize to a birthweight centile, 18.8% of babies at HA were born <10th centile versus 14.3% babies at the LA, Bylakuppe site.

These data support previously identified findings that being born to a woman of Tibetan ancestry in Leh resulted in the highest birthweight for babies. This effect was also found to be enhanced at a LA, with babies born to women of Tibetan ancestry in Tso Jhe Khangsar in Bylakuppe South India (850 m a.s.l.) found to have an average birthweight of 3.5 kg (pre-term <37 weeks of 2.9 kg; table 1). Stillbirth rates in Leh reached 25.2/1000 births compared with 5.2 in the UK over the same time period and 9.7 across India, according to the national family health survey (NFHS) in 2016 (https://dhsprogram.com/pubs/pdf/fr339/fr339.pdf).

**Table 1.** Comparison of the birthweights of two populations of similar ancestry at HA (Leh) and LA (Bylakuppe), taking into account gestational age and excluding cases where this could not be established (Leh *n* = 34, Bylakuppe *n* = 32).

	Term (>37 weeks gestation)	Preterm (<37 weeks gestation)
**Leh 3500 m**		
Number of participants	305	41
Birthweight (kg)	3.04 (1.60−4.20)	2.11 (0.90−3.00)
Gestation (days)	270 (260−294)	240 (213−258)

**Bylakuppe 800 m**		
Number of participants	64	6
Birthweight (kg)	3.50 (2.00−4.40)	2.90 (2.67−2.99)
Gestation (days)	280 (260−308)	231 (175−256)
	*p* = 0.0001; 95% CI −0.5874 to −0.3248	

### (a) HAPS phenotype data

The HAPS study reported on 316 pregnant women recruited in Leh and 101 in Delhi [19]. More than 96% of women recruited in Leh considered themselves of Ladakhi descent (with a five generation or more documented history of living at altitude). Only 10 (3.2%) described themselves as Tibetan, although birthweight was greatest in this group (3.62 kg) versus Ladakhi (3.14 kg), *p* 0.0009.

Maternal parity, smoking status, chronic illness and socio-economic factors were not statistically different between women who delivered babies born <10th centile small for gestational age (SGA) versus appropriately grown (AGA) babies. However, compared with women who delivered an AGA baby, those who delivered an SGA baby were statistically older and lighter.

UtA diameters, measured at 18−22 weeks, were significantly smaller in women who went on to have SGA babies, compared with AGA. This difference was not seen in the LA Delhi population. A multivariable logistical regression model was run to incorporate potentially interacting factors and included maternal body mass index and age. The UtA diameter remained statistically different between women who had an SGA baby versus those who had an AGA baby (OR 1.06; *p* = 0.0001), with increased maternal UtA positively associated with having an AGA baby.

### (b) HAPS genetic analysis

**Figure 1.**
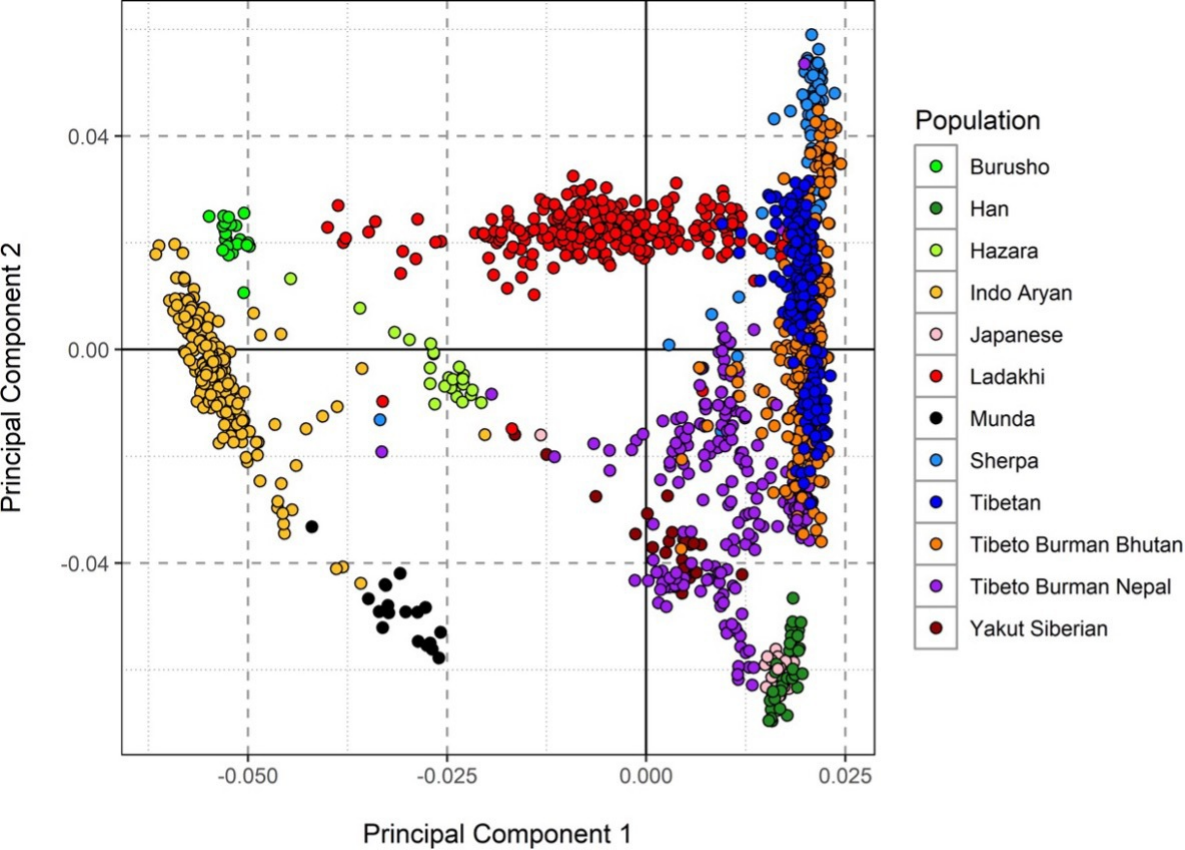
PCA of Ladakhi versus neighbouring populations (Ladakhi in red). Reproduced from [20].

Genetic analysis showed that the Ladakhi population were more closely related to Tibeto-Burman-speaking populations than to the Indo-Aryan groups of South Asia, with the majority forming a common clustering Principle Component Analysis (PCA), indicating the presence of a Ladakhi-specific genetic ancestry component, intermediate between Tibeto-Burman and Indo-Aryan ancestries, which ADMIXTURE analysis supported (figure 1).

### (c) Birthweight analysis

We determined whether there were any genome-wide significant predictors of birthweight in the Ladakhi population, performing a Genone Wide Association Study of birthweight on the 316 neonates recruited. We did not find any signals that reached genome-wide significance after correction. However, when we looked in the tail of the association statistics, we noted the presence of variants associated with haematological traits of potential relevance to HA adaptation, including rs16893892 in *RP5-874C20.3,* which is associated with mean corpuscular haemoglobin; rs2298839 in *AFP* associated with mean platelet volume and rs9261425 in *TRIM31* associated with white blood cell count.

Analysis of overlapping genetic signals observed in relation to recognized birthweight signals at LA were also investigated in this population. Overall, 32 of these 70 recognized signals [28] were either genotyped or captured through linkage disequilibrium (*r*^2^ > 0.8) in the Ladakhi dataset. Seven of these 32 signals were significantly associated (*p* < 0.05, uncorrected) with birthweight from our Global Screening Array dataset [29].

## 4. Discussion

The Ladakh population provides a new dimension to HA research. One of the added values of this population is the careful phenotyping of family trios (pregnant woman, partner and newborn) in association with the availability of genotype data. Over the course of the HAPS research project more than 500 families were approached and recruited, with many trios providing both phenotype data and biological samples for genotyping and other metabolic studies.

The data generated by HAPS have updated the information available about pregnancy outcomes specific to the Ladakhi population, providing a more structured review of the level of adaptation that this population exhibits in response to long-standing HA ancestry. Birthwight was most protected in babies born to women of Tibetan ancestry. However, the data also supported a protective, albeit weaker protective effect on birthweight in the ancestrally recognized Ladakhi pregnant population when gestational age was taken into account.

Similar birthweight trends have been identified in other HA cohorts. A retrospective review of a cohort of South American birthweights reported a 3.15 kg average birthweight for HA Andean (longest adapted) subjects, compared with their European (shortest) counterparts at HA at 2.96 kg [1]. The absence of retained significance of most classically recognized maternal risk factors in our multivariable model is also in keeping with earlier work based on Colorado HA residents, which identified HA as an independent risk factor influencing birthweight [2].

Appropriately grown babies at HA in Leh are both heavier and longer than their counterparts born at LA, suggesting that intrinsic mechanisms that influence body size and composition may have a role to play in positive adaptation at HA. Equally, our UtA diameter results replicate previously reported greater UtA blood flow findings (owing to diameter, not flow velocity) seen at both weeks 20 and 36 of pregnancy in Andean versus European women [30]. Animal studies of the mechanisms underpinning UtA vasodilation support a role for the metabolic sensor, AMPK, which was found to be present in placental tissue and influenced vasodilation in the presence of chronic hypoxia [31–33]. Given that the UtA phenotype in the Leh cohort validated previous HA cohort findings [8,30], further work is planned to identify possible therapeutic interventions in pathways associated with UtA dilatation that might benefit fetuses *in utero*.

Interestingly, stillbirth rates in Leh in this period remained significantly higher than the national average at the time. Stillbirth may be seen as the ultimate adverse consequence of poor growth, but it remains to be seen whether other factors may also have been contributing in the population. We could not take into account all factors related to fetal growth and stillbirth risks in the analysis. It is possible that factors such as previous obstetric history or recurrence risk of diseases) or differing standards or approaches to antenatal and delivery care at each of the sites, might have impacted birthweight differently and influenced the increased incidence of stillbirth seen in the Ladakh population.

Genetic results suggest that a considerable component of the contemporary Ladakhi genome has descended from ancestral highlander populations that have resided on the Tibetan plateau for the last 35 000 years. There has been subsequent admixture with neighbouring Indo-European populations but the majority of the Ladakhi population form their own distinct cluster. The analysis performed provides evidence for where overlap can be sought between other HA populations but also that there may be unique physiological, environmental and genetic adaptations in this population that can be best explored through detailed study of the population.

Physiological study of pregnant women, their partners and newborns in Leh, compared with a LA Delhi population has shown some fundamental differences [23]. Intriguingly, physical differences in the Ladakh population, compared with others in India, were identified through the more detailed work carried out during HAPS. These include features such as dietary differences (more meat eating practices in Ladakh, with 96% of recruited women eating meat versus 41% in Delhi) as well as physical feature differences in Ladakhi pregnant women, whose SGA babies were heavier and taller compared with women who had SGA babies in Delhi. These factors need to be considered when identifying and associating relevant factors protecting birthweight at HA but also when considering genetic adaptation being recognized in specific relation to birthweight as a physiological outcome. *HMGA2*, which is the gene encoding the high mobility group-A2 protein and one of the SNPs that replicated in the Ladakh population, has been associated with human height and birthweight in lowland populations [34]. *ZBTB38*, another SNP that was revealed, encodes a zinc finger transcription factor and its expression appears to play a role in skeletal development [35] and human height [36]. The association with gene variants that confer advantage in skeletal, metabolic and height parameters warrant further exploration, especially in relation to functional correlation with probable higher birthweight. This is an area in which further research is planned.

In summary, the Ladakhi population represents an ancestrally ancient HA population, similar to but distinct from the Tibetans. The presence of a hospital in Leh, with a mainly institutionalized (i.e. hospital-based) delivery rate makes for a relevant and accessible study population. Initial data support the existence of an adaptive process that protects birthweights. However, births that are small for their gestational age and stillbirths remain a challenge and comparison between this population and those born at a healthy birthweight might be useful when defining the physical and genetic features of hypoxia adaptative processes.

## Data Availability

All relevant data are within the manuscript.
